# Using simulation-based learning to provide interprofessional education in diabetes to nutrition and dietetics and exercise physiology students through telehealth

**DOI:** 10.1186/s41077-019-0116-7

**Published:** 2019-12-20

**Authors:** Marie-Claire O’Shea, Nathan E. Reeves, Andrea Bialocerkowski, Elizabeth Cardell

**Affiliations:** School of Allied Health Sciences, Griffith University, Gold Coast Campus, Southport, QLD 4222 Australia

**Keywords:** Student, Simulation-based learning, Dietitian, Exercise physiologist, Interprofessional education, Diabetes, Simulated patient

## Abstract

**Background:**

Current workforce demands require new graduates to competently work within health care teams and often in remote settings. To better prepare students for this work, universities have spent much time developing interprofessional education (IPE) activities. The body of literature supporting IPE of allied health students is growing. Simulation-based learning with simulated patients is one platform through which IPE can be implemented in a dedicated, supported environment and potentially at scale. This study describes an interprofessional simulation-based learning experience with nutrition and dietetics and exercise physiology students. The common practice area of interacting with patients who have type 2 diabetes was targeted, and the simulation was delivered in partnership with simulated patients via a telehealth platform to allow interprofessional teams to work collaboratively in remote locations.

**Methods:**

Ten nutrition and dietetics and 13 exercise physiology students participated in a simulation module in which students observed and collaborated in the development and delivery of an interprofessional treatment plan for patients with diabetes. Learning outcomes were measured according to the first two levels of Kirkpatrick’s (1994) model for training evaluation (i.e. reaction and learning), as well as the perceived impact on behaviour.

**Results:**

The students’ confidence in communication, assessment, management and ability to work with another health professional significantly increased (*p* < 0.05) post-activity. Students perceived that the simulation-based learning would have a positive impact on their clinical skills and ability to work with other health professionals. Students reported that the most effective aspects of the simulation module were learning from and about each other, the opportunity for experiential learning and the supportive learning environment. However, the telehealth platform audio clarity and delay had negative impact on the learning experiences for students.

**Conclusion:**

The overall positive results demonstrate the potential of simulation-based learning activities for preparing allied health students for working in interprofessional teams. Although remote access was possible, the telehealth platform was identified as a limiting factor to this simulation-based learning experience. However, videoconferencing technology has advanced considerably since this study. Hence, there is an opportunity to employ more reliable technology for future simulations.

## Background

Interprofessional education (IPE), also referred to as interprofessional learning, forms an integral part of curriculum planning and teaching activities with universities as they respond to the World Health Organisation’s Framework for Action on Interprofessional Education and Collaborative Practice [1]. Whilst many different definitions of IPE can be identified in the literature, the authors of this study have adopted the Centre for the Advancement of Interprofessional Education definition; ‘Interprofessional Education occurs when two or more professions learn with, from and about each other to improve collaboration and the quality of care’ [2]*.*

Increasingly, the literature documents the importance of IPE in the healthcare setting. Emerging in the literature are improvements in patient health outcomes, satisfaction [3, 4], safety, compliance, cost of care and morbidity when interprofessional collaborative practice is undertaken [3–5].

The Australian Government has demonstrated its commitment and support of an interprofessional workforce through the funding of projects, pilots and initiatives and subsidising primary IPE healthcare schemes. One such example is the nationally funded scheme which ‘enables General Practitioners to plan and coordinate the care of patients with complex conditions requiring ongoing care from a multidisciplinary team’ [6].

In order to staff the workforce with appropriately trained ‘interprofessional practice-ready’ health professionals, student training in IPE is paramount [5]. In Australia, IPE leaders from higher education and the health sector have identified areas of IPE pedagogy which include a set of interprofessional ‘capabilities that are meaningful and relevant’ across all health practice areas [7]. Terms such as ‘graduate capabilities’ [5, 8] and ‘competency domains’ [5, 8, 9] are widely used as tertiary education institutions work to embed IPE within health professional curricula.

- The literature provides a broad range of ideas and concepts for developing interprofessional learning activities. Common IPE activities include:
- Participation in tutorials, in-services and combined treatment sessions during student placement programs, involving actual patients attending consultations within the University clinic [10]
- Large-group workshops including multi-professional lectures and problem-based learning cases [11, 12]
- Small group simulations involving a simulated patient or role play or modelling activities undertaken within groups [13–18]

Evidence indicates that the inclusion of simulated patients (SP) within IPE simulation is increasing, and is beneficial to students [19]. The term SP has been defined by Barrows as ‘a normal person who has been carefully coached or trained to portray a specific patient’ [20]. This term has been further developed and modernised by Bearman and Nestel in 2015; ‘a simulated participant, who may at times be called upon to standardise their portrayal’ [21]. Literature indicates IPE activities including SPs have several clear benefits over other activities such as role plays, for example, increased simulation fidelity by increased authenticity, provision of standardised and therefore equitable experiences for each participant and the capacity for immediate feedback to participants from the trained SPs [22].

This growing body of evidence to support IPE activities within health education is largely in relation to medicine and nursing [23], and there is little published data available exclusively relating to interprofessional activities between allied health professionals. A 2007 systematic review of IPE activities in health education found that most activities occurred between medical and nursing students, with other allied health such as pharmacy and occupational therapy appearing less often. Of the 21 identified studies, nutrition and dietetics (N&D) and exercise physiology (EP) did not appear at all [24]. A 2012 review by Abu-Rishand colleagues found only one study to include non-medical and non-nursing cohorts [25].

In their systematic review specifically targeting IPE in allied health, Olson and Bialocerkowski [17] stated that medical-based IPE should ‘not be assumed to be transferable into allied health curricula’, noting that there are large differences and professional paradigms in service delivery models and teaching principles [26]. The authors contended that further investigation, implementation and careful evaluation and review of non-medical-based IPE was now warranted.

In 2008, the governing bodies of Australian dietitians and exercise physiologists established a Joint Position Statement aimed at ‘improving collaboration between the professions, enhancing mutual respect and understanding, and advancing patient outcomes’ [11]. This position statement recognised the benefits of collaborative and coordinated approaches to patient care and, as one example, formalises the need for each discipline’s unique skill set in the treatment of patients with type 2 diabetes. In preparing students for work readiness, it is clear that training and experience in interprofessional care is required. The workforce has already established the interrelated nature of N&D and EP in patient care, therefore providing IPE experiences to students across these disciplines seems logical and required.

IPE through simulation and in partnership with SPs continues to expand into different aspects of student training including the emerging area of telehealth [27]. An emerging and necessary modality of healthcare delivery, telehealth has been pinned to boosting productivity, improving access to healthcare and to overcoming workforce shortages in rural and remote communities [27–29].

Curriculum and accreditation requirements for N&D and EP programs do not specify the need for telehealth training, possibly leaving graduates underprepared for the workforce. However, an innovative example of a university telehealth training program is the Simulated Telemedicine Environment Project for Students (STEPS) funded by the Australian Department of Health. The primary aim of the STEPS project was to ‘increase the quality and quantity of clinical education’ [30]. This saw the development and implementation of multidisciplinary telehealth simulation-based learning experience (SBLE) for students across the disciplines of physiotherapy, speech pathology, dietetics, exercise physiology, nursing and pharmacy.

This paper reports on collaboration between the nutrition and dietetics and exercise physiology disciplines in developing, implementing and evaluating a SBLE for students. Focusing on the management and assessment of a patient with type 2 diabetes, the simulation aimed to provide a supported environment for development of discipline-specific skills and interprofessional collaboration. The simulation also provided simulation designers new to interprofessional collaboration, an opportunity to explore and develop skills in this area.

Given that a telehealth platform was being trialed, a final aim was to introduce students to telehealth consultations in preparation for real practice. The SBLE is reported in alignment with the published ‘Reporting Guidelines for Health Care Simulation Research: Extensions to the CONSORT and STROBE Statements’ by Cheng et al. [31]. In reporting our findings, we will be adding to the body of evidence around the value of SBLE and IPE simulation for allied health students and seek to provide recommendations for future practice.

## Method

This study received ethical approval from the Griffith University Human Research committee (GU Ref No: PES/40/12/HREC). Written informed consent was obtained from all participants.

### Participants

A total of ten nutrition and dietetics students and 13 exercise physiology students participated in the simulation. The participant details, including experience with simulation and videoconferencing, are detailed in Table 1.

**Table 1:** Experience of student participants

Student type	Gender	Age group		History of SBLE		Videoconference experience	
		20–24	25–29	No. of sessions		No. of sessions	
				Range	Mean	Range	Mean
N&D	M = 1; F = 9	4	6	0-1	< 1	0	0
EP	M = 6; F = 7	8	5	1-2	1	0-1	< 1

*N&D* Nutrition and dietetics, *EP* Exercise physiology

The simulation was designed by one discipline lead from nutrition and dietetics and one from exercise physiology. Both had training and experience in simulation design however held novice skills in IPE simulation. The facilitators were trained and experienced in providing student feedback however had not provided facilitation in an IPE or telehealth setting prior to this activity.

### Setting and context

The simulation modules were conducted on campus at Griffith University. Clinic-consulting rooms and adjoining waiting area in the student-led Health Clinic were used to create an authentic clinical environment for the simulation. Students were either face-to-face with the SP or consulted via the telehealth platform. The module was embedded into the N&D curriculum in the penultimate year of a 4-year bachelor program, prior to final practical placement. For EP students, the module was the third in a series of five SBLE across the 1 year post graduate diploma program. The entire cohort of the N&D and EP program participated in the simulation. Prior to the simulation activity, all students were required to complete the scenario pre-reading via their course online learning site.

### The telehealth system

**Fig. 1:**
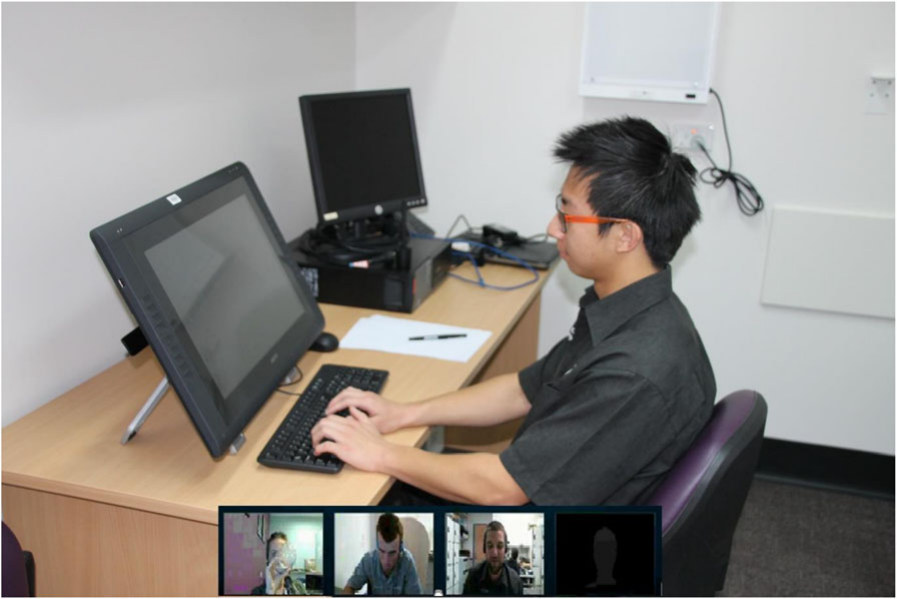
Students’ view of the screen during the simulation

Following investigation and consultation, WebEx® (Cisco WebEx, Milpitas, CA https://www.webex.com.au/) was the selected web-based videoconferencing platform. The intent was to replicate industry current practice for telehealth service delivery and case conferences. Figure 1 shows an image of the students’ view of the screen during the simulation.

The facilitator was able to view each student pair (i.e. one N&D student and one EP student) and the patient (i.e. an employed SP). The students and SPs could only view the consultation interaction as the facilitator video was deliberately deactivated to increase fidelity. The facilitators and SPs received orientation to the case and the Webex system prior to the activity.

### The simulation module

The simulation module targeted diabetes and aimed to provide students with the opportunity to observe a student from another profession conduct a patient consultation and then collaborate with each other in the development and delivery of an interprofessional treatment plan (see Table 2).

**Table 2:** Simulation case details and student learning objectives

Case study • 60-year-old male, newly diagnosed type 2 diabetes • Appointments held at the ‘clinic’ • EP student: Follow up appointment to provide home exercise program • N&D student: Initial appointment (diet history, physical screening and diet plan)	
Learning objectives • Described the role of an EP/Dietitian in supporting a person to manage type 2 diabetes • Discuss how EP and Dietitians can work together to support a person to manage type 2 diabetes • Develop a management plan for a person with type 2 diabetes actively participate in a reflective debrief discussion	

**Fig. 2:**
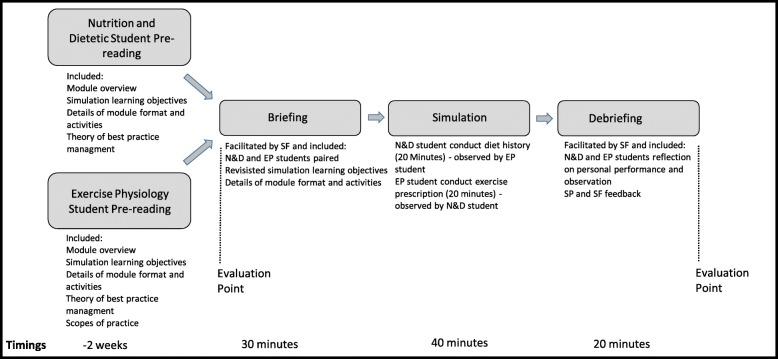
Summary of simulation activity

A pre-simulation online component included the professional scope of practice of each discipline and Joint Position Statements [32], best practice treatment guidelines for people with type 2 diabetes, theoretical underpinnings of motivational interviewing specific to patients with type 2 diabetes, example case study scenario and details of the case to be used in the simulation (including referral from medical practitioner, Health Summary sheet and Exercise Physiology Initial Assessment Report). On the day of the simulation event, each student group participated in an orientation and briefing. Figure 2 provides an overview of the simulation including time and activities. The EP student presented their pre-prepared exercise program to the N&D student and provided an evidenced-based rationale for the prescription. The N&D student presented on their approach to conducting a diet history with the scenario case. The 20-min simulation activity saw the EP student deliver an exercise programming consultation to an SP whilst the N&D student observed. The N&D student then conducted a diet history with an SP whilst the EP student observed. A debrief was conducted immediately following the simulation activity. The SP, facilitator and students provided feedback with self-reflection encouraged. At the discretion of the facilitator, the debrief either followed an informal structure that was triggered by observations made of the students or a more scripted formatted which included four key questions; ‘how effectively did you meet the goals you set for this appointment?’, ‘what did you observe the other student do that was unexpected?’, ‘how well did you collaborate with the other students?’ and ‘what is the take home learning that will influence your future practice?’

### Evaluation and analysis

Student perception of the SBLE was measured pre- and post-simulation activity on Kirkpatrick’s (1994) first two levels of training evaluation that is reaction and learning. Reaction was evaluated pre- and post-simulation through the intrinsic motivation inventory (IMI) [31]. The IMI is a 7-point scale to assess learners’ subjective experience related to 7-subscales of interest, competence, tension, usefulness, choice, importance and relatedness. The IMI can be modified to suit specific environments, hence this study focused on four relevant sub-scales only; investigate student interest, competence, tension and usefulness of a given task. Learning was evaluated post-simulation through a series of 5-point Likert scale questions (1 = strongly disagree and 5 = strongly agree). These were developed by the simulation designers and based on the activity learning outcomes that is, the students’ perceived impact on clinical performance in the four activity areas of communication, assessment, management, and IPE. Open-ended questions also were included (see Table 3).

**Table 3:** Post-simulation questions to evaluate learning via perceived impact on clinical performance

Rate your perceived impact that the addition of simulation-based learning may have on your: 1. Performance in the area of communication 2. Performance in the area of client assessments 3. Ability to work with another health professional 4. The simulation has made me better prepared to manage a patient with diabetes in collaboration with another health professional 5. Performance in the area of client management (completed by EP students only as this was not a learning outcome specific to N&D students)	
Open ended responses required: Please describe the most effective part of the simulated learning experience that positively impacted on your clinical performance during this clinical placement. Please describe the least effective part of the simulated learning experience that did not positively impact on your clinical performance during this clinical placement. How could this be improved?	

All statistical analyses were completed using SPSS version 22 (SPSS Inc., Chicago, IL). Wilcox sign rank test was used to compare disciplines and Mann-Whitney *U* text to compare scores pre and post simulation. Median score and interquartile range (IQR) were also reported for each discipline and for each variable.

## Results

### Quantitative findings

A total of 23 students (N&D = 10 or 100%, EP = 13 or 100%) responded to the post survey.

The following key findings emerged:

1. Overall, both cohorts’ perceptions of the outcomes and benefits of the SBLE was high (medians ranged from 4.00 to 5.00 on the 5-point scale), with 90% responding in agreement or strong agreement to each statement.
2. Students in both disciplines perceived competence post-simulation was significantly higher than pre-simulation perceived competence (N&D pre-median 3.72 and post median 4.86 on a 7-point scale; *p* = 0.21, EP pre-median 4.43 and post median 6.0 on a 7-point scale; *p =* 0.001).
3. The N&D students had significantly higher pre-simulation levels of tension compared to the EP students (median of 5.00 and IQR of 4.20–5.40 vs. 4.00 and 3.20–4.20 for N&D vs. EP; *p* = 0.04). Post-simulation, perceived levels of tension were lower than pre-simulation for both disciplines (N&D median of 4.10 and IQR of 3.60–5.20, EO median 3.20 and 1.40–4.40); however this was only significant for EP (*p* = .032).
4. One hundred percent of students from both cohorts perceived that the SBLE would have a positive impact on their clinical performance in the areas of communication and their ability to work with another health professional. Performance in the area of assessment was still reported positively by most students (90% of N&D students and 91.7% of EP students).

A total of 15 students (EP = 9 or 69%, N&D = 6 or 60%) responded to the open-ended questions on the most and least effective aspects of the SBLE (Table 4). All responses were reviewed by two authors and key themes derived. Any conflicts or discrepancies were resolved through discussion by the two authors. Responses were similar for both disciplines and reported positive aspects of the simulation, resulting in four themes: learning from and about others, experiential learning, learning environment and improvement and least effective aspects of the SBLE.

**Table 4:** Qualitative comments reported by students

	Total respondents	Themes	Examples of students’ comments
Q1 Learning from and about others	5 (N&D = 3) (EP = 2)	Being able to impart knowledge to other students.	‘Being able to see how other students, from my own profession as well as others, interact with an actor…’ (N&D Student 1). ‘Ability to understand a dietitian role’ (EP Student 11)
Q2 Experiential learning	5 (N&D = 1) (EP = 4)	Practicing their skills in a clinical environment	‘Having the practice of going through the procedure with a patient.’ (N&D Student 2). ‘Always done initial assessments so this one was good in that we were able to go through the next stage with a client.’ (EP Student 9).
Q3 Learning environment	4 (N&D = 2) (EP = 2)	Working with an actor, the inclusion of debriefing, and the safe learning environment.	‘It was a safe environment to make mistakes and ask questions about clinical stuff.’ (N&D Student 10).
Q4 Improvement	2 (N&D = 1) (EP = 1)	Improvement in their own skills leading to increased confidence positively impacted on their learning.	‘Improving my communication and confidence.’ (EP Student 4).
Q5 Least effective aspects	9 (N&D =4) (EP = 5)	Students suggested changes to timing on the day, timing within teaching semester, the need for more preparation and difficulties with the videoconferencing clarity.	‘I needed more time to prepare for the consultation.’ ‘The video conferencing system was not always easy-to-use, or the sound wasn’t clear enough.’

## Discussion

This SBLE provided students with the opportunity to collaborate in an important practice area in a supported environment. This case study provides a valuable addition to the limited published literature of simulation-based learning activities within exercise physiology and nutrition and dietetics education [23]. It also demonstrates the ability to utilise simulation-based learning within an IPE pedagogy and embed IPE within health professional curricula.

The results indicate that students successfully worked alongside and with each other to provide input and feedback on treatment aspects and plan of intervention strategies for the patient. The new and valuable learning that took place from, with and about each other [2] was evident in the evaluation data through increased perceptions of confidence, competence and interest following the simulation, as well as increased knowledge about the other profession’s scope and role.

There was significant learning that took place by the simulation designers. Key factors emerged from the experience and should be considered by others before embarking on an interprofessional SBLE:

1. *Clear and early identification of discipline specific learning objectives.* It is possible to have varying levels of practical experience amongst the students. In this case, N&D students were participating in their first SBLE and had almost no previous experience counselling a patient. The EP students were f5 weeks from completing their last placement and degree, which somewhat explains their higher pre-simulation confidence. Despite this difference in ability level, the simulation module still met the learning objectives of each discipline and the IPE objectives were similar for both.
2. *Experienced facilitators to design and lead orientation and debriefing.* Training and prior simulation experience of the simulation designers enabled this module to meet the recommended design characteristics as described by Jeffries [33] and Arthur [34]. Experienced facilitators are more likely to be aware of the importance of flexibility when running simulation activities. For example, student numbers can vary at any stage due to changes in timetables, availability and illness. A single student change may result in major timetable and group allocation changes, causing additional stress to the untrained or inexperienced simulation facilitator. The designers of this simulation strongly believe that the continued success of this SBLE is contributed to by having experienced facilitators.
3. *A well*-*developed evaluation plan that utilises common or frequently used tools in contemporary simulation research.* The present simulation was well evaluated. However, new tools and evaluation frameworks have subsequently emerged that can measure the different levels of learning. These should be considered in future simulations.

### Limitations of the simulation design

The simulation module had two learning objectives; IPE collaboration and telehealth service delivery training. Despite both outcomes being achieved, changes to funding meant that the telehealth component of the SBLE was no longer a requirement. It became apparent to the simulation designers that the time, effort and costs relating to the telehealth component (which included a technology support person) would not be sustainable. Furthermore, the evaluation data strongly supported IPE collaboration and very easily met the discipline specific learning objectives. A decision to plan the subsequent year’s simulation module without the telehealth component was agreed upon.

A second limitation was the lack of follow-though to ascertain the impact of the simulation on students’ future behaviours around IPE, telehealth and practice more generally. Running focus groups would have provided some insights, here.

## Conclusion

### Future IPE simulation activities

Since this initial simulation module in 2014, four more iterations have been conducted. Non-telehealth simulations began in 2015 and only minor modifications have occurred during this time to accommodate variable student numbers, access to simulation venues and budget considerations. Changes to the evaluation components were required as it became increasingly evident that the Kirkpatrick’s model had limitations [35]. During the 2017 simulation, a self-designed evaluation tool using the previously mentioned Joint Position Statement [32] was implemented to analyse the collaborative outcomes of the SBLE. This data is yet to be analysed.

### Future research

Future research includes comparison of the simulation activity against current ‘best practice guidelines’ by the International Nursing Association for Clinical Simulation and Learning ‘Standards of Best Practice: Simulation^SM^’ [36]. The next stage of evaluation involves analysis of student assessment scores post simulation module, perceived impact of this simulation module on placement preparedness and ultimately, evaluating the impact of the simulation module on graduate practice.

As the authors of this paper have done, future research should be reported based on Cheng’s guidelines [31]. A concerted and collaborative effort to raise the standard of simulation reporting will enable the body of evidence to underpin curriculum design based on evidence-based pedagogy within the disciplines of nutrition and dietetics and exercise physiology.

## Data Availability

The datasets used and/or analysed during the current study are available from the corresponding author on reasonable request.

## Abbreviations

EP: Exercise physiology
IPE: Interprofessional education
N&D: Nutrition and dietetics
SBLE: Simulation-based learning experience
SP: Simulated patient
STEPS: Simulated Telemedicine Environment Project for Students
